# How Does Servant Leadership Nurture Nurses' Job Embeddedness? Uncovering Sequential Mediation of Psychological Contract Fulfillment and Psychological Ownership

**DOI:** 10.1155/2023/7294334

**Published:** 2023-02-21

**Authors:** Naveed Ahmad Faraz, Zhengde Xiong, Sultan Adal Mehmood, Fawad Ahmed, Khansa Pervaiz

**Affiliations:** ^1^Business School, Hunan University, Changsha, Hunan, China; ^2^Department of Management Sciences, University of Jhang, Jhang, Pakistan; ^3^Entrepreneur College, Xi'an Jiaotong-Liverpool University, Suzhou, Jiangsu, China

## Abstract

**Aim:**

This research aimed to explore how servant leadership nurtures nurses' job embeddedness by uncovering the sequential mediation of psychological contract fulfillment and psychological ownership.

**Background:**

The healthcare of Pakistan is undergoing an acute shortage of 1.3 million nurses. The gap is widening due to unprecedented natural uncertainties (floods, earthquakes, COVID-19, dengue, polio, and monkeypox) and the large-scale brain drain of nurses. Therefore, exploring the underlying factors that could facilitate nurses' job embeddedness is imperative.

**Methods:**

A cross-sectional research design was employed, wherein data were gathered in three rounds, two months apart, from 587 nurses employed in public hospitals in Pakistan, and analysis was performed with Smart-PLS.

**Results:**

Servant leadership positively influences nurses' job embeddedness and psychological contract fulfillment. Besides, psychological contract fulfillment positively affects psychological ownership, and psychological ownership enhances nurses' job embeddedness. Finally, psychological contract fulfillment and psychological ownership sequentially mediate the relationship between servant leadership and job embeddedness.

**Conclusions:**

This research emphasized the vitality of servant leadership in nurturing nurses' job embeddedness. *Implications for Nursing Management*. Healthcare authorities should keenly focus on promoting servant leadership that shapes the positive perception of nurses about their psychological contract fulfillment and psychological ownership, which are essential resources to cherish nurses' job embeddedness.

## 1. Introduction

According to recent estimates by the World Health Organization (WHO), there will be a need for 36 million nurses practicing around the globe by 2030. The report highlighted that achieving the sustainable development goal of health and well-being would require 13 million additional nurses and midwives. Furthermore, a report titled “sustain and retain in 2022 and beyond” also revealed how the COVID-19 pandemic had worsened the fragile state of the global nursing workforce, putting the World Health Organization's aim of Universal Health Coverage at serious risk. Healthcare experts anticipate a migration tsunami more than ever before. In addition, the preexisting unequal distribution of nurses worldwide will be exacerbated by large-scale international recruitment to high-income countries, which will only widen inequalities in access to healthcare globally. There is already a substantial scarcity of professional nurses in developing countries [1]. For instance, in Pakistan alone, there is a shortage of 1.3 million nurses, and the gap is widening due to unprecedented natural uncertainties (floods, earthquakes, COVID-19, dengue, polio, and monkeypox) and the large-scale brain drain of nurses. Therefore, exploring the underlying factors that could facilitate the retention of nurses in developing countries is imperative to overcome these grave issues of shortage of nurses and their brain drain.

The scholarship on employee retention has shifted its focus to why employees want to stay with an organization instead of leaving [2]. Primarily, this shift is the outcome of job embeddedness theory [3], which describes three critical components of employees' job embeddedness, including links, fit, and sacrifices that shape their decisions to remain tied with an organization. In this context, employees' job embeddedness is a broader and more meaningful construct than employee turnover and has garnered scholarly interest in the organizational behavior literature owing to its vitality in shaping various positive outcomes and reducing negative impacts. Extant research establishes its instrumentality for reducing turnover, work performance, organizational citizenship behavior [4], career success [5], perceived self-leadership [6], affective commitment, and voice behavior [7]. However, healthcare, especially nursing, is a relatively nuanced research context in job embeddedness research and what leads to nurses' job embeddedness from the perspective of the conservation of resources theory (COR). The COR theory posits that individuals are always motivated toward resource generation and accumulation [8]. Resource investment is one of the basic principles of COR, which accentuate that individuals tend to exploit available resources, such as servant leadership, to protect against loss of resources, recover the lost resources, and gain more resources in the future [9]. This study intends to employ the COR theory as an overarching theoretical framework to unravel the underlying process that nurtures job embeddedness in healthcare.

Extant research has established the pivotal role of leadership in shaping employees' attitudes and behaviors [1, 10]. Among the value-based leadership styles, servant leadership has been considered more employee-centric, resulting in valuable employee outcomes. Greenleaf [11] coined the servant leadership approach that accentuates grooming followers to their fullest potential. Servant leadership believes in improving individuals' lives and focuses on creating better organizations and a caring world [12]. A recent literature review of leadership in healthcare by [13] considered the scarcity of empirical research exploring the effectiveness and benefits of servant leadership. In response to their research call, this study posits that servant leadership will positively influence nurses' job embeddedness.

From the employees' perspective, job embeddedness is regarded as the state of resource abundance, i.e., the desired state where employees accumulate affective and nonaffective job-related constraints (links, fit, and sacrifice), which enmesh employees in a specific job within a specific organization [14]. However, research is yet to unlock the underlying process of servant leadership, perceived as a resource, in realizing job embeddedness, i.e., the state of resource prosperity. Apropos of this interesting literature gap, drawing on COR theory, we advance that employees' psychological contract fulfillment and psychological ownership would be the mediating channel through which nurses realize job embeddedness. Employees' psychological contract fulfillment represents their perception of fairness in executing the agreed commitment, promises, and terms and conditions made in the employment relationship by the employer [15]. However, in an organizational context, employees' psychological ownership describes their psychological state wherein they feel possession of the organization's material or nonmaterial artifacts [16]. Servant leadership, an employee-focused leadership style, is believed to positively influence nurses' job embeddedness through employees' psychological contract fulfillment and psychological ownership.

Our study has primarily three objectives: (1) to explore the direct influence of servant leadership on nurses' job embeddedness through the lens of COR theory; (2) to offer nuanced theorizing of COR in explaining the underlying phenomenon of psychological contract fulfillment and psychological ownership in the relationship between servant leadership and nurses' job embeddedness; (3) to respond to the call for research to unravel the effectiveness of servant leadership in healthcare. This research contributes to the literature on servant leadership and job embeddedness in three distinctive ways: (1) It uniquely employs the underpinnings of the COR theory in explaining the nuanced relationship between servant leadership and nurses' job embeddedness through the sequential intervening mechanisms of psychological contract fulfillment and psychological ownership. Doing so offers the first empirical evidence to advance the COR theory in this domain. (2) It empirically supplements the rarely examined direct relationships between servant leadership and psychological contract fulfillment, psychological contract fulfillment and psychological ownership, and psychological ownership with job embeddedness. (3) It responds to calls for a scholarship to examine the effectiveness of servant leadership within healthcare (James et al., 2021) and to explore the intervening psychological mechanisms of servant leadership in predicting employees' outcomes [1].

## 2. Theoretical Background and Hypotheses

### 2.1. Servant Leadership and Job Embeddedness

Job embeddedness encompasses the perceptual and contextual factors that retain employees in location, jobs, colleagues, and the organization [17]. Mitchell et al. [3] refer to job embeddedness as a social web comprised of primarily three factors: (i) links, whether the formal or informal association of an employee with colleagues, (ii) fit, representing the degree to which employees' job aligns with other aspects of their lives, and (iii) sacrifice, referring to the anticipated cost of psychological or physical benefits given up by quitting a job. Primarily, job embeddedness focuses on the three earlier enlisted aspects that retain employees in their jobs. For instance, more links cultivated by employees will result in significant expenses, whether psychological, emotional, or financial [3]. Likewise, when employees' knowledge, skills, and abilities coincide with their jobs and find opportunities for personal and professional growth, they will feel a greater sense of fit with the job [18]. Lastly, sacrifice may refer to material costs (cost of relocation) or psychological cost (perceived psychological contract fulfillment, and job security) or the cost of benefits associated with a particular job [18].

Greenleaf [11], who introduced the concept of servant leadership, argued that motivation to serve others and subsiding personal gains over followers' well-being must be servant leaders' core considerations [19]. He was concerned with making the world a better place, requiring organizations to fulfill the needs of their employees and the least privileged in society. He firmly believes that those being led should benefit from the leaders, or at least not further harm. Greenleaf accentuated that the decisive role of servant leadership is to equip the followers personally and professionally and inculcate servanthood in them to create a better world by serving others [11].

The concept of servant leadership as a value-based leadership style has garnered scholarly interest owing to its vitality in shaping employees' attitudinal and behavioral outcomes [20]. Extant research establishes its instrumentality over a wide range of outcomes, including employees' performance, organizational citizenship behavior, nurses' burnout, psychological well-being, and many more (for details, see the systematic literature review of Eva et al. [21]. Nevertheless, despite the vitality of servant leadership in healthcare, there is a scarcity of empirical research exploring its effectiveness (James et al., 2021). In response, this study posits that servant leadership would positively influence nurses' job embeddedness. Furthermore, though servant leadership and job embeddedness are prevalent areas of research interest among scholars and practitioners [4, 22], the linkage of these two concepts is inadequately investigated in the existing literature [21], especially since there is not a single study on this relationship in healthcare.

Servant leadership focuses on followers' personal and professional development, which aligns with the “fit” facet of job embeddedness. Servant leaders are determined to satisfy their staff's needs and leverage their full support to resolve personal and occupational problems. In addition, servant leaders' support and positive association with nurses create a close bond of trust and respect [1]. Similarly, servant leaders follow an open-door policy and establish close one-on-one relationships with their subordinates, thus creating a congenial working environment that facilitates the “link” dimension of job embeddedness. Besides, subordinates' formal and informal connections with leaders and peers uplift their sense of attachment and belongingness. Lastly, nurses would perceive a high value in the sacrifice of leaving a job in the presence of servant leaders because such leaders are considered as most concerned about the well-being of followers, trustworthy, and caring about the psychological contract fulfillment of employees.

From a theoretical perspective, the limited research on the relationship between servant leadership and job embeddedness primarily relied on social exchange theory and found a positive relationship [4]. However, for deeper insights, scholarship invites alternate theoretical frameworks, for instance, the conservation of resources (COR) theory [21], to unravel the underlying process of servant leadership in shaping nurses' job embeddedness.

Building on COR theory [8], we contend that the facets of servant leadership [19]: emotional healing, empowering, putting followers first, ethical behavior, relationship building, and servanthood supplement the essentials of the job embeddedness, i.e., links, fit, and sacrifice, and nurses' never want to lose these valuable resources that will presumably be further invested to gain more resources [9]. This discussion led us to postulate the following.

**Hypothesis 1 .**

Servant leadership has a positive influence on nurses' job embeddedness.

### 2.2. Servant Leadership and Psychological Contract Fulfillment

A psychological contract refers to “individual beliefs, shaped by the organization, regarding terms of an exchange agreement between individuals and their organization” [15]. Employees' psychological contract fulfillment reflects the extent to which their employers satisfy implied commitments made in the employment relationship [15]. Though a psychological contract comprises the supposed commitments of an employer (organization) with the employee, the employer is undoubtedly represented by someone, primarily the organizational leadership, in its relationship with the employee [23]. Certainly, immediate managers or supervisors are seen as the “chief agent for establishing and maintaining the psychological contract” [23].

Servant leadership emphasizes nurturing the well-being of all the stakeholders, and it would be interesting to unravel its influence towards employees' psychological contract fulfillment, which gauges the cognitive evaluation of well-being in the employment relationship. We contend that servant leadership is the most suited leadership style for fulfilling employees' psychological contracts because employees' needs, interests, and growth are at the core of this leadership philosophy. Servant leaders put employees first and are so concerned about their well-being that they prioritize employees above the organization's benefits. Such leaders convince the organization's decision-makers to invest resources in uplifting employees' knowledge, skills, and abilities. In addition, they act as a bridge between the organization and employees to abide by contractual promises.

Then, the distinguished characteristics of servant leadership [19] like sensitivity towards concerns of employees, facilitation in resolving their issues, building long-term relationships, demonstrating genuine concern for their career growth, and serving them even through self-sacrifice are all such pious traits that positively shape employees' perceptions of their psychological contract with the organization.

The limited empirical evidence [23], primarily employed social exchange theory, establishes a positive influence of servant leadership on employees' psychological contract fulfillment. Therefore, other theoretical explanations are valuable to substantiate the existing findings to fill this void in the literature. Extending this line of thinking and grounding this discussion within the theoretical underpinnings of the COR theory [9], it is posited that servant leaders' investment in opportunities like offering training, leveraging operational skills, career growth, mentoring, and job security would be positively perceived as resource gain by the nurses, which strengthens psychological contract fulfillment. Thus, for the reasons mentioned earlier, the following is hypothesized.

**Hypothesis 2 .**

Servant leadership has a positive influence on nurses' psychological contract fulfillment.

### 2.3. Psychological Contract Fulfillment and Psychological Ownership

Psychological ownership, at its core, comprises a sense of possession or the feeling that an individual owns an object [24]. Generally, psychological ownership is viewed through the lens of the individual-physical object relationship. However, it is a broader concept that may include nonphysical objects like artistic creations, ideas, and organizations. Our operationalization of psychological ownership focuses on nonphysical psychological ownership, representing employees' attitudinal state where they become attached to and intentionally invest themselves in an organization.

Broadly speaking, there are two types of psychological contracts, namely, transactional psychological contracts and relational psychological contracts. First, transactional psychological contracts are temporary trade-offs that may subside the development of psychological ownership. In contrast, relational psychological contracts have the potential to foster long-term organizational psychological ownership. Such contracts are relationship-oriented and require a substantial emotional and social investment and attachment of the employee and employer [25].

Griep et al. [26] identified that little is known about psychological contract fulfillment's role in cultivating employees' psychological ownership. It is argued that when servant leadership strengthens employees' psychological contract fulfillment, the latter serves as the basis for realizing unconscious psychological affection for the organization, i.e., psychological ownership [27]. Servant leaders represent the face of the organization to employees and invest valued resources in employees to satisfy their unconscious needs. The fulfillment of psychological contracts builds a positive image of an organization among its employees, and they perceive it as a resource worth having. We advance that psychological contract fulfillment strengthens the employees' belief that their organization is reliable in fulfilling its promises [28]. In return, employees start forming stronger relationships with the organization. They start investing their time, ideas, resources, abilities, intellect, and physical and psychological energies into the organization. Anchoring the lens of COR theory, which accentuates that individuals invest resources for future gains [9], we advance that assurance of psychological contract fulfillment strengthens employees' belief that the organization is reliable enough to invest resources in the long run. Empirical evidence supports our postulation. For instance, the study in [29] found a positive association between psychological contract fulfillment and psychological ownership. This discussion leads us to frame the following.

**Hypothesis 3 .**

Nurses' psychological contract fulfillment has a positive influence on their psychological ownership.

### 2.4. Psychological Ownership and Job Embeddedness

From a border perspective, psychological ownership and job embeddedness represent psychological states where employees own and desire to continue with an organization. According to COR theory, these psychological states of employees are considered to be the desired condition of resourcefulness and may act as a buffer against resource loss [9]. The process that nurtures psychological ownership in the organizational context would also elevate employees' job embeddedness. For instance, a better understanding of organizational culture, procedures, values, goals, and skills will develop a person's psychological ownership and increase his compatibility with the organization. The COR theory advances that people are motivated to accumulate resources [9] and invest their available resources to gain more. Considering job embeddedness as the target, employees will invest their time, effort, and energy or strengthen links within the organization to be compatible with the job. In either case, there is a strong likelihood of boosting job embeddedness. Job embeddedness, a state of resource abundance, would attract employees to invest their existing resources, such as psychological ownership, to multiply the valued resources. Backing these arguments empirically, the findings of a study by Mehmood et al. [14] revealed a positive association between psychological ownership towards organizational embeddedness. Thus, to advance this narrative, the following is posited:

**Hypothesis 4 .**

Nurses' psychological ownership has a positive influence on their job embeddedness.

### 2.5. Sequential Mediation of Psychological Contract Fulfillment and Psychological Ownership

The conceptualization of “resources” is central to the COR theory [8]. Halbesleben et al. [30] defined resources as anything perceived by an individual to hold value capable of helping to attain the desired objectives. Accordingly, the value of a resource increases when an individual perceives it as helpful to achieve a goal when he or she already possesses it and when there is a new resource that complements it [30]. In the preceding sections, passageways have been identified that lead to employees' job embeddedness, a state of resource abundance. Hobfoll et al. [9] conceived that resources do not exist in isolation but instead travel in caravans or packs for organizations and individuals. They further introduced the concept of resource caravan passageways that accentuated that individuals' resources exist in ecological conditions that either foster and nurture or limit and block resource creation and sustenance. For instance, servant leadership support, psychological contract fulfillment, organizational ownership, and job embeddedness are highly interrelated and rooted in similar environmental conditions.

Consistent with Wheeler et al. [31], we conceptualized job embeddedness as a resource caravan representing diverse types of resources (i.e., fit, links, and sacrifices) with the potential to supplement each other. In advancing our propositions, servant leadership offers instrumental support in encouraging employees to realize the state of resourcefulness, in this case, job embeddedness. We contend our argument by proposing that servant leadership creates job embeddedness by triggering a resource gain spiral in the form of psychological contract fulfillment and psychological ownership. Precisely, servant leadership facilities psychological contract fulfillment that nourishes the roots (efficacy, self-identity, and belonging) and routes (control, investment of self, and intimate knowledge of the target) of psychological ownership [16], which boosts job embeddedness. Thus, grounding our contemplation in the underpinnings of the COR theory, it is advanced that employees' positive perception of servant leadership initiates the resource caravan that travels through psychological contract fulfillment, uplifting organizational ownership, and shaping job embeddedness, a milestone of resource abundance. Therefore, the following is hypothesized.

**Hypothesis 5 .**

Nurses' psychological contract fulfillment and psychological ownership sequentially mediate the positive influence of servant leadership on nurses' job embeddedness.

**Figure 1:**
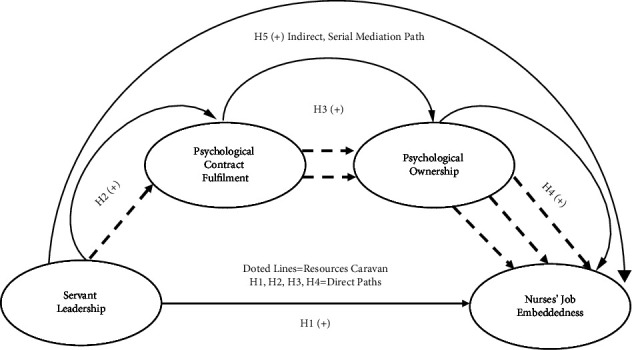
Theoretical model.

Figure 1 presents the theoretical model of this study.

## 3. Methods and Measures

### 3.1. Samples and Procedures

This study targeted the nurses employed in the public hospitals of Pakistan. A total of 10 hospitals were shortlisted, two operating in each provincial capital and the country's federal capital, having the maximum number of nurses and also serving as nursing schools/colleges. The number of beds in the selected hospitals ranged between 3000 and 4000, and the number of nurses was between 1023 and 1472. The HR department of the shortlisted hospitals was briefed about the objective of the research, explained that participation is voluntary where the anonymity of the participants will be ensured, and sought permission to approach the nurses about participating in the surveys. The lists containing information about nurse ID, name, and e-mail address were obtained from the HR departments of the selected hospitals. By employing systematic random sampling, 100 nurses from each hospital, accounting for 1000 nurses, were shortlisted for survey participation. The authors designed three survey forms on Google Docs to capture the independent variable, mediating variables, and dependent variable at three different time instances to establish causality and overcome the common method bias [32]. Multiwave online surveys, two months apart, were carried out from January to June 2022. During the first half of January 2022, an initial survey containing questions about demographics and immediate supervisors' servant leadership was administered to 1000 nurses. The first wave yielded 762 responses. After two months, during the second half of March 2022, a questionnaire containing questions about psychological contract fulfillment and psychological ownership was administered to 762 nurses who had participated in the initial survey. The second wave yielded 649 responses. The third survey was performed in the first half of June 2022, wherein 649 nurses who had participated in the previous two waves were asked to respond to questions on job embeddedness. The outcome of the third wave was 587 complete responses. There were no missing values, as surveys were designed to restrict nonresponsiveness.  Table 1 summarizes the demographics of the participating nurses.

### 3.2. Measures

All measures employed in this research were adopted from reputable studies and have proven validity and reliability. The five-point Likert scale (five = “strongly agree” and one = “strongly disagree”) was introduced to capture responses from the nurses. Responses about servant leadership were obtained through a 7-item global servant leadership measure designed by Liden et al. [19]. Researchers widely employ this measure, which has proven validity and reliability (*α* = 0.89). The illustrative item contains “My leader puts my best interests ahead of his/her own.” Psychological contract fulfillment was gauged by adopting 4-item measure from a recent study by Yu [33], who adapted these items from the original measure of psychological contract breach [25]. Cronbach's alpha of the revised measure was 0.89, and a sample item includes “So far, my employer has done an excellent job of fulfilling its promises to me.” Then, the psychological ownership of nurses was evaluated by a 7-item measure advanced by Van Dyne and Pierce [34]. The reliability coefficient of the scale is 0.90, and one of the items narrates, “I sense that this is my company.” Lastly, the study employed a 9-item measure to examine nurses' job embeddedness [35]. The measure comprised three dimensions, namely, links, fit, and sacrifice. The overall reliability of the measure was 0.88, and a sample item comprised “My job utilizes my skills and talents well.”

## 4. Data Analysis

The data analysis was carried out using the partial least squares path modeling (PLS-PM) method through the Smart-PLS version 4.0.8.3 application. We prefer PLS-PM because of the following: (i) It is better suited for research having a prediction-oriented nature [36]. (ii) It eases analysis for research having many constructs with multiple indicators and hypothesized paths [37]. (iii) Specifically, it is the preferred technique for sequential mediation analysis [38]. (iv) In the recent decade, it has proven its dominance of application in research in the filed human resource management [37]. (v) It comprised advanced metrics and thresholds for the assessment of the measurement model and structural model and comparatively, offers improved statistical power [36]. The data analysis in PLS-PM is performed in two distinctive stages, which are presented in detail in the subsequent sections.

### 4.1. Evaluation of the Measurement Model

Confirmatory composite analysis (CCA) is an effective procedure for estimating the PLS-PM measurement model [39]. Job embeddedness is the only reflective-reflective second-order construct, while all the other variables in this study are reflective first-order constructs. The evaluation of the measurement model is performed through the following four phases.

During the first phase, the reliability of the individual items of constructs was evaluated through the items' loadings, ensuring that the loading values were greater than or equal to 0.708, the recommended threshold value [39]. Then, the second phase deals with establishing the reliability of all the variables at the construct level. The recommended metrics for gauging the construct's reliability are Cronbach's alpha and composite reliability, and the threshold range of these metrics is between 0.70 and 0.95 [36]. In the third phase, the potential issue of convergent validity is addressed by employing the commonly used statistic of average variance extracted (AVE) metric [36]. The recommended value of the AVE metric should be greater than 0.50, describing that the individual items have explained 50% or more of the variance in the overall construct. The factor loadings of all the items, Cronbach's alpha, composite reliability, and average variance extracted are listed in Table 2, ensuring the evaluation of the measurement model as per the recommended thresholds.

The fourth phase of the measurement model evaluation revolves around confirming the discriminant validity among the constructs. In the PLS-PM technique, it is strongly suggested to employ the heterotrait-monotrait (HTMT) ratio of correlations whose desired threshold is less than 0.90 [36]. Statistics establishing the discriminant validity of constructs are presented in Table 3.

### 4.2. Evaluation of the Structural Model

The latest developments and guidelines on PLS-SEM are followed while assessing the structural model, and the steps carried out during that assessment are narrated in the subsequent paragraphs.

The first step of the structural model is to fix the potential issue of multicollinearity, where the variance inflation factor (VIF) is the most commonly used metric. Therefore, the VIF values of all the indicators of the constructs under consideration are listed in Table 2, which are within the recommended limit, i.e., below 03 [39].

The hypothesized relationships are evaluated in the second step through multiple metrics, including the path coefficient (*β*), standard error, *t*-values, *p* values, and bias-corrected confidence intervals. The bootstrapping procedure of the Smart-PLS was employed to obtain the values of the earlier said metrics. Hair et al. [36] recommended the use of percentile bootstrap confidence intervals for analyzing the significance of a specific hypothesized path. As a rule, the intervals should not contain a zero value for statistical significance. The results of the hypothesized relationships are presented in Table 4.

**Figure 2:**
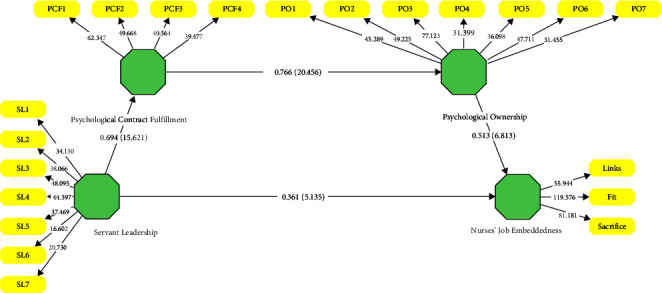
Structural model results.

The final step of the structural model assessment offers an assessment of the coefficient of determination (*R*^2^), values that predict the percentage variance explained for all the endogenous variables of the structural model. The threshold values of the coefficient of determination are 0.25, 0.50, and 0.77, which describe the variance predicted in the endogenous construct as weak, moderate, and substantial, respectively [36]. The values of the coefficient of determination (*R*^2^) are listed in Table 4. Finally, Figure 2 presents the results of the assessment of the structural model.

## 5. Discussion and Implications

### 5.1. Discussion

Through this research, the authors intended to unravel the influence of servant leadership on nurturing nurses' job embeddedness and explore the sequential mediation of psychological contract fulfillment and psychological ownership. Building on the theoretical underpinnings of the COR theory [9], the present study has proposed resource caravans and passageways, which are crucial to embedding nurses in their jobs.

At first, it was contemplated that servant leadership has a direct positive influence on nurses' job embeddedness, which is endorsed by the findings of this study (H1: *β* = 0.361, *t*-value = 05.135, *p* value < 0.05, and confidence interval without having zero value). The rationale behind this finding is grounded in the COR theory [9], which emphasizes that individuals strive for valuable resources. Servant leaders offer followers a blend of resources, including social, positional, and organizational [1] which are essential to uplift the necessary components of links, fit, and sacrifice of job embeddedness. Servant leadership is built on compassionate love [40], wherein the well-being and care of the followers are of primary importance. Leaders with servant leadership value each individual and build cordial working relationships with their subordinates. They actively listen to their problems and leave no stone unturned to excavate them from problems. All the pious characteristics of servant leadership work closely with the essentials of job embeddedness, i.e., links, fit, and sacrifice. Therefore, it can be claimed that nurses working under servant leaders never want to lose these valuable resources and embed them in their jobs. Extant empirical scholarship examining the influence of various leadership styles on job embeddedness is scarce, although emerging. Our finding is consistent with the limited research available on this discourse [4, 41, 42].

Secondly, it was postulated that servant leadership would uplift nurses' psychological contract fulfillment. Results have lent credence to this claim (H2: *β* = 0.694, *t*-value = 15.621, *p* value < 0.05, and confidence interval without having zero value) and are in line with the limited available research. Our literature review found two studies that examined the influence of servant leadership in strengthening employees' psychological contract fulfillment and their findings lent credence to this relationship [23, 43]. In addition, research also establishes a negative association between servant leadership and psychological contract breach [44], a construct to some extent the opposite of contract fulfillment. Servant leadership pays special attention to each subordinate's unique potential and inspires trust, guides, offers feedback, and provides resources to realize his/her potential to grow and succeed [21]. Being very well aware of their followers' needs, servant leaders selflessly serve their followers and build close, trustworthy relationships [40]. Such leaders always put their subordinates first through their words and actions and always behave pretty, honestly, and transparently [19], reinforcing their credibility in front of nurses and thus satisfying their psychological contract fulfillment.

The third hypothesis that psychological contract fulfillment positively shapes psychological ownership has been proven (H3: *β* = 0.766, *t*-value = 20.456, *p* value < 0.05, and confidence interval without having zero value) and is consistent with existing research [29]. Contrary to psychological contract fulfillment, scholarship has identified that employees' perception of psychological contract breach adversely impacts their psychological ownership and may lead to adverse behavioral outcomes [45, 46]. Assurance of psychological contract fulfillment gives a green signal to nurses about the organization's credibility, and they start owning the organization.

Fourth, psychological ownership was supposed to influence nurses' job embeddedness positively. The findings of this hypothesis (H4: *β* = 0.513, *t*-value = 06.813, *p* value < 0.05, and confidence interval without having zero value) support the extant research on this relationship [14]. Gardner et al. [29] did examine the positive influence of psychological contract fulfillment toward job-based psychological ownership in shaping citizenship behavior among employees in China. Nurses with psychological ownership see them with the organization for an extended time. They invest that ownership to gain more resources in the form of job embeddedness [9].

Lastly, results also support the sequential mediation of psychological contract fulfillment and psychological ownership in the relationship between servant leadership and nurses' job embeddedness. In the existing literature, few studies have attempted to explore the intervening role individually played by either psychological contract fulfillment or psychological ownership in predicting employees' outcomes [17, 47]. However, our study was one of the first attempts to unravel the sequential mediation of psychological contract fulfillment and psychological ownership. The results of sequential mediation (H5: *β* = 0.272, *t*-value = 05.452, *p* value < 0.05, and confidence interval without having zero value) can be explained through the conservation of resource theory lens [8]. It is argued that servant leaders' authentic and empathetic behavior strengthens nurses' perceived psychological contract fulfillment, which adds value by shaping nurses' psychological ownership, ultimately leading to enhanced job embeddedness.

### 5.2. Implications for Nursing Management

Numerous practical implications for nursing management can be derived from the results of this study. At first, it unravels a complete process of servant leadership, through which it nurtures nurses' job embeddedness. Unprecedented uncertainties (floods, earthquakes, COVID-19, dengue, polio, and monkeypox) have worsened the fragile state of the global nursing workforce, and the situation is even critical in developing countries. Our study highlighted the vitality of servant leadership in healthcare to cherish nurses' job embeddedness, a valued resource having the potential to deal with the prevailing chaotic situation in healthcare. It can be further inferred that servant leadership in healthcare would facilitate resolving the grave shortage of nurses and their brain drain through job embeddedness. In addition, healthcare should focus on attracting, developing, and retaining nurse supervisors who believe in and act in accordance with the servant leadership philosophy. Besides, the HR department should invest in training, workshops, and seminars to facilitate the workforce in building servant leadership characteristics.

Second, the findings of this study reaffirm the contention that financial incentives are not the only sources to invigorate nurses' perceived psychological contract fulfillment. Other nonfinancial resources, like supportive leadership, can also shape psychological contract fulfillment. Accordingly, authorities in healthcare should keenly focus on alternate intrinsic channels that uplift nurses' psychological contract fulfillment.

Third, our results endorsed that nurses' psychological contract fulfillment is vital in shaping their positive attitudes and behaviors. One of the positive outcomes is psychological ownership. Therefore, nursing management should draft policies and contracts by prioritizing the needs of nurses and should not promise things that could not be fulfilled later.

Last, our findings strengthen the applicability of the COR theory in healthcare. Health workers want to achieve a state of resourcefulness, like job embeddedness. Top management in healthcare is now responsible for providing nurses with a conducive environment, like servant leadership, which will shape their positive perception about psychological contract fulfillment, strengthening the feelings of ownership and resulting in job embeddedness.

### 5.3. Research Limitations and Future Directions

Perfection is usually hard to achieve, in line with scholarship in management science, and this research has a few shortcomings. First, at the outset, the cross-sectional design of this study restrains the possibility of drawing cause-effect predictions from these results. Although authors designed multivalve data collection to have limited causality among variables, it would be wise to have a longitudinal research design in the future for more profound casual predictions. Second, there was single-source data collection in this study more prone to socially desired responses by the participants. This social desirability issue can be addressed in future research by gathering data from diverse primary sources like managers and colleagues and secondary sources like annual turnover reports. Third, psychological contract fulfillment and psychological ownership were included as the sequential mediators in this research, whereas there are other potential mediators like psychological empowerment, psychological safety, organizational justice, and trust in the leader that can be explored in future investigations. Fourth, this research operationalized job embeddedness as a higher-order construct. Aspirant researchers should conceptualize job embeddedness as a lower-order construct for parsimonious findings. Last, we suggest examining whether the nurses, supervised by the servant leaders, have themselves grown as servant leaders or otherwise.

## Figures and Tables

**Table 1:** Demographics of the participants.

Gender	Male	Female		
	09% (53)	91% (534)		

Age (in years)	18–30	31–40	41–50	Above 50
	34% (200)	27% (158)	23% (135)	16% (94)

Years of experience	01–10	11–20	21–30	Above 30
	26% (153)	38% (223)	22% (129)	14% (82)

Level of education	Diploma	Bachelors	Masters	Above masters
	16% (94)	53% (311)	25% (147)	06% (35)

**Table 2:** Evaluation of the measurement model.

Constructs	Items	Loadings	VIF
First-order reflective constructs			
Servant leadership (*α* = 0.913, CR = 0.920, AVE = 0.623)	SL1	0.805	2.623
	SL2	0.819	1.196
	SL3	0.798	1.564
	SL4	0.782	2.279
	SL5	0.818	2.904
	SL6	0.734	2.075
	SL7	0.764	2.376
Psychological contract fulfillment (*α* = 0.821, CR = 0.892, AVE = 0.675)	PSF1	0.812	1.524
	PSF2	0.829	1.837
	PSF3	0.793	2.489
	PSF4	0.851	2.634
Psychological ownership (*α* = 0.917, CR = 0.933, AVE = 0.665)	PO1	0.803	2.482
	PO2	0.839	2.528
	PO3	0.817	2.347
	PO4	0.791	2.367
	PO5	0.803	2.138
	PO6	0.814	2.851
	PO7	0.842	2.068
Job links (*α* = 0.823, CR = 0.835, AVE = 0.629)	JL1	0.793	2.020
	JL2	0.826	2.332
	JL3	0.758	2.307
Job fit (*α* = 0.847, CR = 0.859, AVE = 0.670)	JF1	0.815	2.138
	JF2	0.797	2.857
	JF3	0.843	1.062
Job sacrifice (*α* = 0.825, CR = 0.851, AVE = 0.656)	JS1	0.809	2.375
	JS2	0.794	2.524
	JS3	0.826	2.830

Second-order reflective-reflective construct			
Nurses' job embeddedness (*α* = 0.919, CR = 0.927, AVE = 0.808)	Links	0.891	2.033
	Fit	0.889	2.699
	Sacrifices	0.917	2.979

*α* = Cronbach's alpha; CR = composite reliability; AVE = average variance extracted; VIF = variance inflation factor.

**Table 3:** Discriminant validity through the HTMT approach.

	Mean	SD	JE	PO	PCF	SL
Nurses' job embeddedness	3.06	0.87				
Psychological ownership	3.58	1.02	0.811			
Psychological contract fulfillment	3.31	0.93	0.845	0.816		
Servant leadership	3.29	0.78	0.771	0.736	0.747	

SD = standard deviation.

**Table 4:** Evaluation of the structural model.

Hypothesized relationships	Path coefficient	SE	*t*-value	Confidence interval		Conclusion
				[LL, UL]		
Direct paths						
H1: SL -> JE	0.361	0.069	05.135	[0.246, 0.479]		Supported
H2: SL -> PCF	0.694	0.057	15.621	[0.609, 0.758]		Supported
H3: PCF -> PO	0.766	0.048	20.456	[0.698, 0.822]		Supported
H4: PO -> JE	0.513	0.023	06.813	[0.387, 0.634]		Supported

Sequential mediation path						
H5: PSL -> PCF -> PO -> JE	0.272	0.011	05.452	[0.197, 0.359]		Supported

Quality criteria of the model (*R*-square adjusted)						
Psychological contract fulfillment = 0.479			Psychological ownership = 0.585		Job embeddedness = 0.646	

*p* < 0.05; LL = lower limit; UL = upper limit; SE = standard error. The confidence intervals and *t*-values were obtained by 5000 bootstraps run at two-tailed significance at 5%.

## Data Availability

The data used to support the findings of this study are available from the corresponding author upon request.
